# Human Bone Marrow Mesenchymal Stem Cell-Derived Hepatocytes Improve the Mouse Liver after Acute Acetaminophen Intoxication by Preventing Progress of Injury

**DOI:** 10.3390/ijms15047004

**Published:** 2014-04-22

**Authors:** Peggy Stock, Sandra Brückner, Sandra Winkler, Matthias M. Dollinger, Bruno Christ

**Affiliations:** 1 Department of Visceral, Transplantation, Thoracic and Vascular Surgery, Applied Molecular Hepatology Laboratory, University Hospital Leipzig, Liebigstraße 21, D-04103 Leipzig, Germany; E-Mails: peggy.stock@medizin.uni-leipzig.de (P.S.); sandra.brueckner@medizin.uni-leipzig.de (S.B.); sandra.winkler@medizin.uni-leipzig.de (S.W.); 2 Clinics for Internal Medicine I, University Hospital Ulm, Albert-Einstein-Allee 23, D-89081 Ulm, Germany; E-Mail: matthias.dollinger@uniklinik-ulm.de

**Keywords:** cell transplantation, human mesenchymal stem cells, acute liver injury, stem cell-derived hepatocytes, hepatocyte differentiation

## Abstract

Mesenchymal stem cells from human bone marrow (hMSC) have the potential to differentiate into hepatocyte-like cells *in vitro* and continue to maintain important hepatocyte functions *in vivo* after transplantation into host mouse livers. Here, hMSC were differentiated into hepatocyte-like cells *in vitro* (hMSC-HC) and transplanted into livers of immunodeficient Pfp/Rag2^−/−^ mice treated with a sublethal dose of acetaminophen (APAP) to induce acute liver injury. APAP induced a time- and dose-dependent damage of perivenous areas of the liver lobule. Serum levels of aspartate aminotransferase (AST) increased to similar levels irrespective of hMSC-HC transplantation. Yet, hMSC-HC resided in the damaged perivenous areas of the liver lobules short-term preventing apoptosis and thus progress of organ destruction. Disturbance of metabolic protein expression was lower in the livers receiving hMSC-HC. Seven weeks after APAP treatment, hepatic injury had completely recovered in groups both with and without hMSC-HC. Clusters of transplanted cells appeared predominantly in the periportal portion of the liver lobule and secreted human albumin featuring a prominent quality of differentiated hepatocytes. Thus, hMSC-HC attenuated the inflammatory response and supported liver regeneration after acute injury induced by acetaminophen. They hence may serve as a novel source of hepatocyte-like cells suitable for cell therapy of acute liver diseases.

## 1. Introduction

There is increasing evidence in the literature suggesting bone marrow as a new source of hepatic progenitor cells [1–7]. While the regenerative potential of haematopoietic stem cells appears rather to depend on fusion between donor cells and recipient hepatocytes [8,9], mesenchymal stem cells (MSC) have the ability to differentiate into a number of organ-specific cell types including hepatocytes [10–14]. Their therapeutic potential is characterised both by repair and regeneration of tissue lesions as well as modulation of the immune response by suppressing excess inflammation and inducing tolerance [15–17]. As shown by our group and others, MSC from bone marrow and adipose tissue are capable of differentiating into hepatocyte-like cells *in vitro* and *in vivo* [18–23]. They do not only express liver-specific genes and feature adult hepatocyte functions, but also integrate into the recipient liver and rescue animals from lethal intoxication caused by various noxes such as CCl_4_ [21–24] or d-galactosamine [25]. Undifferentiated MSC appear more resistant against a highly toxic environment and might be better suited for the treatment of acute liver failure [23,25,26]. *Vice versa* MSC pre-differentiated into hepatocyte-like cells efficiently repopulate the recipient liver, and thus seem more eligible to treat chronic diseases such as monogenetic liver diseases [27,28]. The good safety record of both hepatocyte and MSC transplantation in pre-clinical and clinical studies further signifies their clinical potential in treating liver diseases [29,30].

Acute liver failure is one of the most prominent hepatic complications due to viral, pharmacological or chemical intoxication with an incidence of more than 40% of cases being caused by acetaminophen (APAP) in the United States and the United Kingdom [31,32]. Acetaminophen is metabolised by the hepatocyte cytochrome P450 enzyme system. APAP overdose leads to depletion of cellular glutathione pools and formation of free radical and reactive oxygen as well as nitrogen species [33–35]. Since the cytochrome P450 enzyme system is predominantly expressed in perivenous hepatocytes of the liver lobule, acetaminophen toxicity initiates inflammation, hepatocyte impairment, and cell death primarily in perivenous regions of the liver. Under massive injury conditions, in which hepatocyte proliferation is impaired, tissue regeneration involves both hepatocytes [36] and hepatic progenitor cells [37]. Clinically, progressive hepatic damage ends with acute liver failure characterised by jaundice, coagulopathy, and encephalopathy leaving orthotopic liver transplantation as the only therapeutic option. In recent years, hepatocyte transplantation has become a versatile alternative to liver transplantation. So far, hepatocyte transplantation to treat acute liver failure has been applied in around 40 cases worldwide [38,39], though is still awaiting convincing success. Novel cell sources such as stem cell-derived hepatocytes may be a good alternative to adult hepatocytes. In fact, recent data in mice and rats showed that mesenchymal stem cells had the potential to rescue animals from fulminant hepatic failure induced by carbontetrachloride or d-galactosamine. This effect is rather due to paracrine anti-inflammatory, anti-apoptotic and pro-proliferative actions than to hepatic integration of and regeneration by the transplanted stem cells, which is very much appreciated in the situation of drug-induced liver injury [23,25,26,40].

Here, we demonstrate in an immunodeficient mouse model of sub-acute liver failure induced by acetaminophen that hMSC-HC after transplantation into the damaged livers contributed to hepatic recovery short-term and integrated long-term providing functional hepatic tissue repair.

## 2. Results

### 2.1. Acute Liver Injury Induced by APAP in Immunodeficient Pfp/Rag^2−/−^ Mice

**Figure 1.**
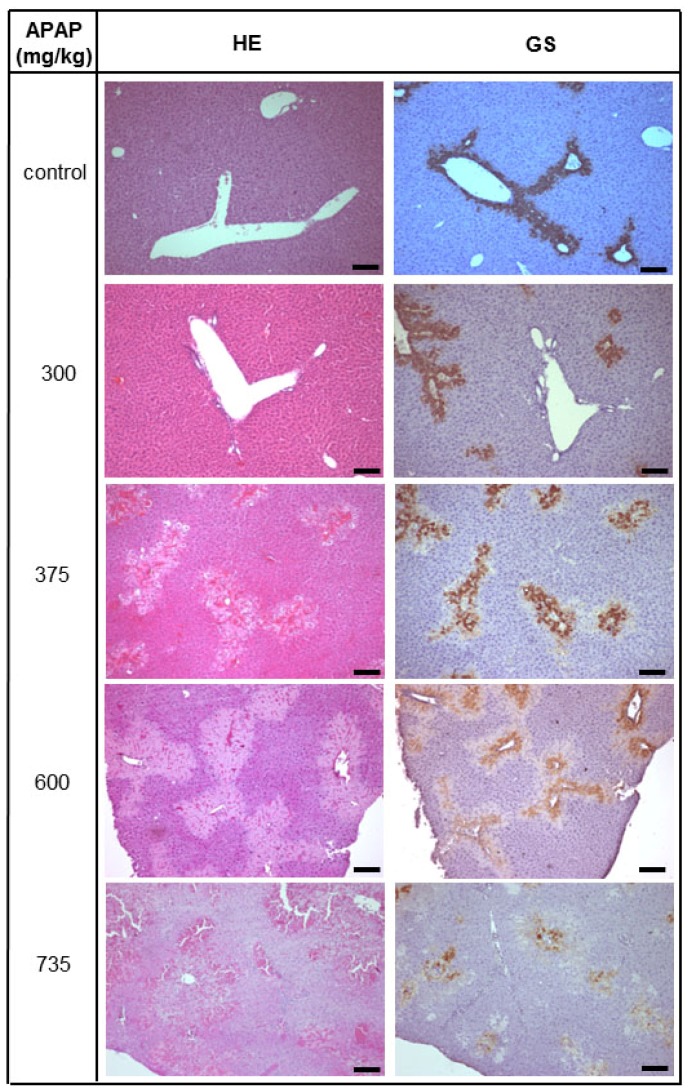
Concentration-dependent damage of mouse livers by acetaminophen (APAP). Animals were injected intraperitoneally acetaminophen dissolved in PBS at the concentrations indicated as described in Materials and Methods. Vehicle controls received PBS. Twenty-four hours later, animals were sacrificed and livers processed for hematoxylin-eosin (HE) staining and immunohistochemical detection of glutamine synthetase (GS) in consecutive tissue sections as described in Materials and Methods. Co-localisation of GS with necrotic areas in the HE stain indicates perivenous deterioration of the tissue, which is typical for intoxication with APAP. Scale bar: 100 μm.

Twenty-four h after treatment, APAP at doses lower than 300 mg/kg body weight did not provoke liver tissue abnormalities. At higher doses, *i.e.*, 375 or 600 mg/kg body weight, APAP caused liver architecture deteriorations in some animals, as expected. However, this damage was not reproducible and occasionally animals treated with these high doses of APAP did not display any liver injury at all. At 735 mg/kg body weight, APAP caused formation of large perivenous necrotic areas as verified by co-staining of glutamine synthetase (GS), which is expressed exclusively in perivenous hepatocytes (Figure 1). At this dose, very few animals died whereas at slightly higher concentrations of 750 mg/kg of APAP more than 90% died. Therefore, in order to induce liver damage by APAP reproducibly without killing the animals, a dose of 735 mg/kg body weight was applied in all subsequent experiments.

In control animals without APAP treatment, serum aspartate aminotransferase (AST) activity was slightly elevated 1 day after partial hepatectomy (2 days *vs.* 1 day after treatment; Table 1). This increase was not changed significantly when hMSC-HC were transplanted after partial hepatectomy. Six days after partial hepatectomy (=7 days after treatment), AST activity returned to initial values again in both settings with and without hMSC-HC transplantation (Table 1, PBS *vs.* PBS + hMSC-HC). Thus, as compared with animals subjected to partial hepatectomy only, transplantation of hMSC-HC did not cause significant additional hepatocyte damage. After 1 day of treatment with 735 mg/kg body weight, APAP increased AST activity about 140-fold. Two days after APAP treatment, in animals not transplanted with hMSC-HC, serum AST was still elevated 18-fold, which then returned to initial levels after another day. Animals receiving hMSC-HC displayed about 3–4-fold higher levels of AST 2 and 3 days after APAP treatment, compared to controls without hMSC-HC. Levels returned to initial values 7 days after treatment (Table 1, APAP *vs.* APAP + hMSC-HC). Obviously, transplantation of hMSC-HC slightly increased tissue damage caused by APAP.

### 2.2. Periportal Localisation of Transplanted hMSC-HC without APAP Treatment

**Figure 2.**
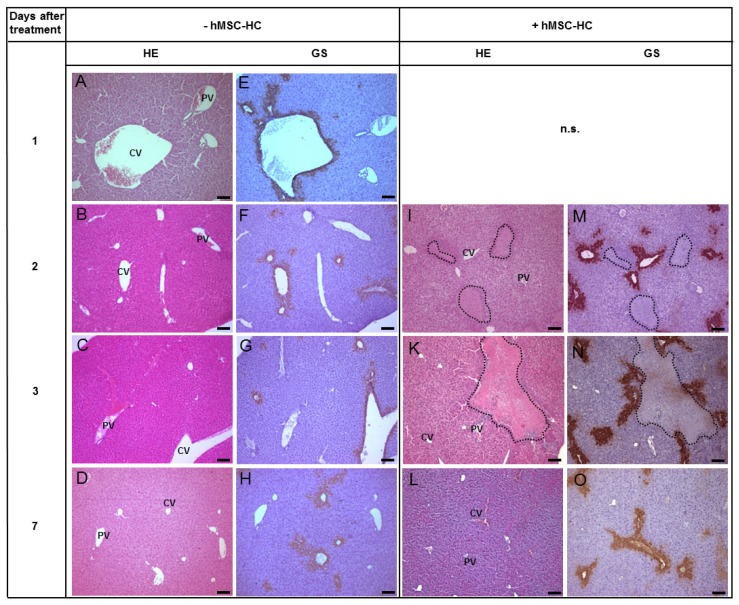
Periportal localisation of transplanted hMSC-HC in livers of mice without APAP treatment. Mice were injected PBS as a control. One day later (=1 day after treatment), one third hepatectomy was conducted and hMSC-HC were delivered via intrasplenic injection (+hMSC-HC) as described in Materials and Methods. Controls received PBS instead of hMSC-HC (−hMSC-MSC). Another 1 day (=2 days after treatment), 2 days (=3 days after treatment) and 6 days (=7 days after treatment) later, livers were harvested and tissue sections stained with HE (**A**–**D**,**I**–**L**); Additionally, consecutive sections were stained for immunohistochemical detection of glutamine synthetase (GS) to localise perivenous areas of the liver (**E**–**H**,**M**–**O**). Dotted lines surround clusters of transplanted cells that are predominantly visible in the periportal regions of the liver. cv, central vein; pv, portal vein; Scale bar: 100 μm; n.s., not shown (same as pictures (**A**,**E**)).

Control animals were injected PBS and 24 h later one third partial hepatectomy was conducted to provide a regenerative stimulus. Subsequently, hMSC-HC or PBS as a vehicle control was administered intrasplenically as described in Materials and Methods. Another 1, 2, and 6 days later, livers were excised and tissue architecture was analysed by HE staining (Figure 2A–D,I–L) and by immunohistochemical staining of glutamine synthetase (GS) (Figure 2E–H,M–O) to localise perivenous areas of the liver lobule. Control animals, undergone partial hepatectomy and injection of PBS instead of hBM-MSC, featured normal liver histology over the time period investigated indicating that the surgical procedure did not change the liver architecture significantly (Figure 2A–H). One and two days after cell transplantation, large clusters of donor hMSC-HC were visible in the periportal areas of the host liver parenchyma (Figure 2I,M,K,N), thus corroborating results from previous studies [20,41]. Six days post-transplantation, the liver histology appeared normal again (Figure 2L,O).

### 2.3. Tissue Distribution of Transplanted hMSC-HC after APAP Treatment

**Figure 3.**
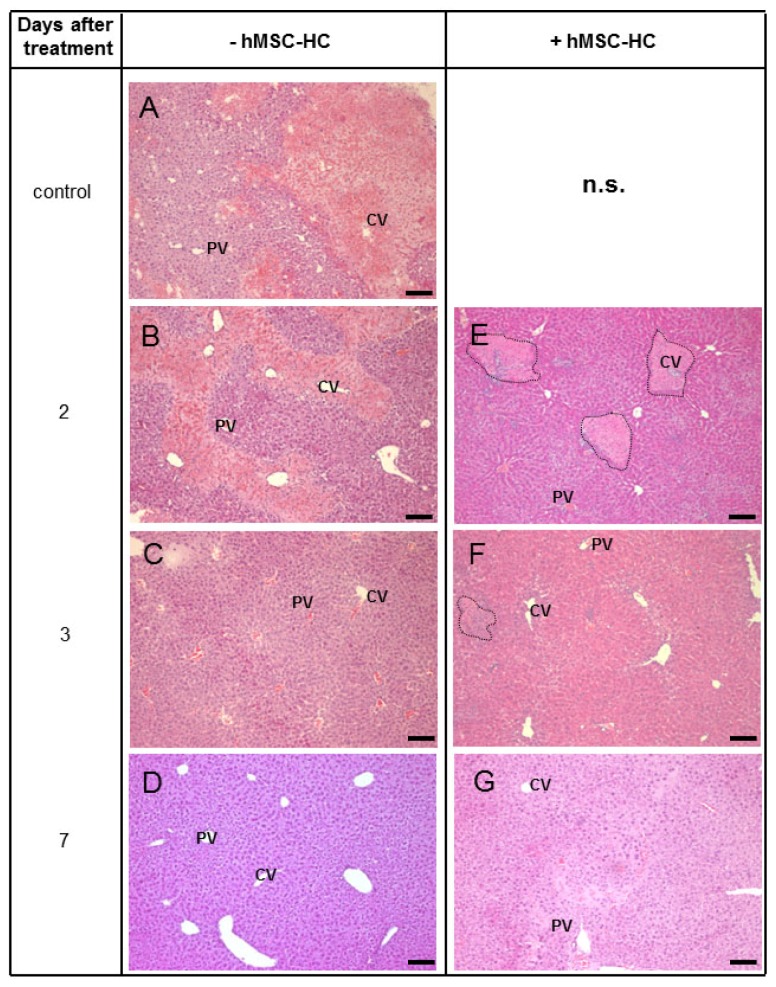
Time course of liver damage after treatment with APAP and hMSC-HC. Mice were treated as described in the legend to Figure 2. One day after APAP treatment, one third hepatectomy was conducted and hMSC-HC were delivered via intrasplenic injection (+hMSC-HC, (**E**–**G**)) as described in Materials and Methods; Controls received PBS instead of hMSC-HC (−hMSC-MSC (**A**–**D**)); Another 1 day (=2 days after treatment (**B**,**E**)), 2 days (=3 days after treatment (**C**,**F**)) and 6 days (=7 days after treatment (**D**,**G**)) later, livers were harvested and tissue sections were stained with HE. Dotted lines enclose clusters of transplanted cells that are predominantly visible in the perivenous regions of the liver. cv, central vein; pv, portal vein; Scale bar: 100 μm; n.s., not shown (same as **A**).

As expected, in livers treated with APAP, 1 (Figure 3A) and 2 (Figure 3B) days after treatment, massive tissue deteriorations were visible, which were primarily located perivenously. Three days (Figure 3C) after APAP treatment, tissue lesions had nearly recovered and were completely absent at day 7 (Figure 3D). Two days after treatment with APAP, hMSC-HC transplants were found in frequent clusters of cells. Surprisingly, these seemed to be localised in the perivenous areas of the liver (Figure 3E,F). Interestingly, no obvious tissue damage was visible in the remaining parenchyma. Seven days after treatment, liver histology seemed normal again and only few remaining clusters of hMSC-HC were occasionally detected (Figure 3G).

### 2.4. Perivenous Localisation of Transplanted hMSC-HC after APAP Treatment

**Figure 4.**
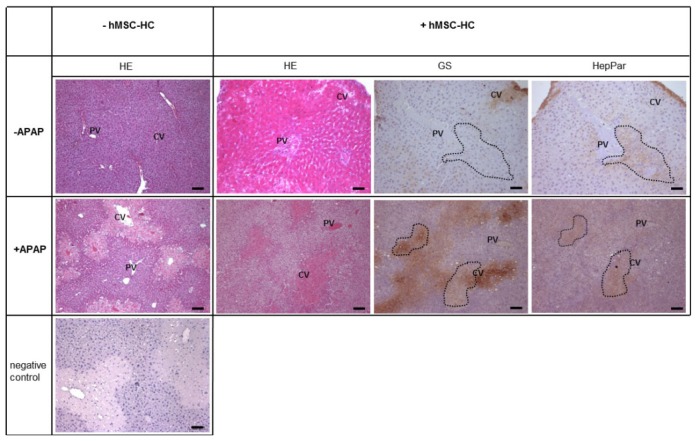
Perivenous localisation of transplanted hMSC-HC in livers of mice treated with APAP. Mice were treated as described in Figure 3. Cells were delivered via intrasplenic injection (+hMSC-HC), control mice received PBS (−hBM-MSC). Three hours later, livers were excised and tissue sections were stained with HE, and consecutive sections were stained for immunohistochemical detection of glutamine synthetase (GS) to localise perivenous areas of the liver as well as for HepPar1 to identify human cell transplants. Dotted lines surround clusters of transplanted cells that are predominantly visible in the periportal areas of untreated livers (upper panels) and in the perivenous regions of the liver after APAP treatment (lower panels), respectively. The bottom left picture shows a negative control staining using the goat anti-mouse IgG secondary antibody, which was combined with the anti-HepPar1 antibody during the specific staining procedure. Comparable results were achieved with all secondary antibodies used as listed in Table 2. cv, central vein, pv, portal vein. Scale bar: 100 μm.

In order to corroborate the unexpected perivenous appearance of transplanted cells after APAP treatment, the localisation was further investigated short-term after transplantation (3 h after transplantation, *i.e.*, 1 day + 3 h after APAP treatment). APAP caused significant tissue lesions in perivenous areas as demonstrated by HE staining (Figure 4, −hMSC-HC). We hypothesized that these damages lead to downstream low pressure regions in the perivenous areas of the liver lobule that might facilitate the passage of transplanted cells from upstream periportal areas of the liver lobule to the damaged perivenous areas (*cf.* Figure 3). Donor cells were identified by the expression of HepPar1 that labelled human cells in the negative mouse liver background. Perivenous areas of the liver were identified by staining of GS. In control livers not treated with APAP, cell transplants were located in periportal areas of the liver as expected (Figure 4, upper panels). This is consistent with the results shown in Figure 3. After treatment with APAP, however, cell transplants were found in the perivenous areas of the liver as shown by co-staining of GS and HepPar1 in consecutive tissue sections (Figure 4, lower panels). Thus, transplanted cells in the short-term range migrated to and colonised the perivenous areas of the liver damaged by APAP.

### 2.5. Attenuation of Hepatocyte Damage and Stimulation of Proliferation by Transplanted hMSC-HC

**Figure 5.**
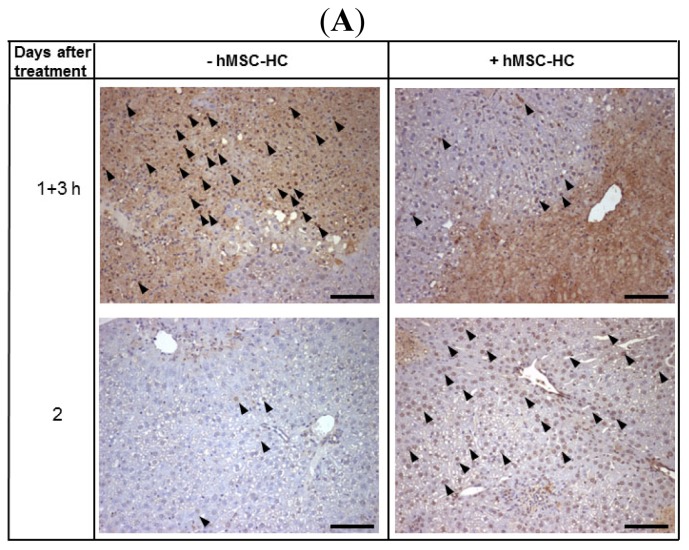

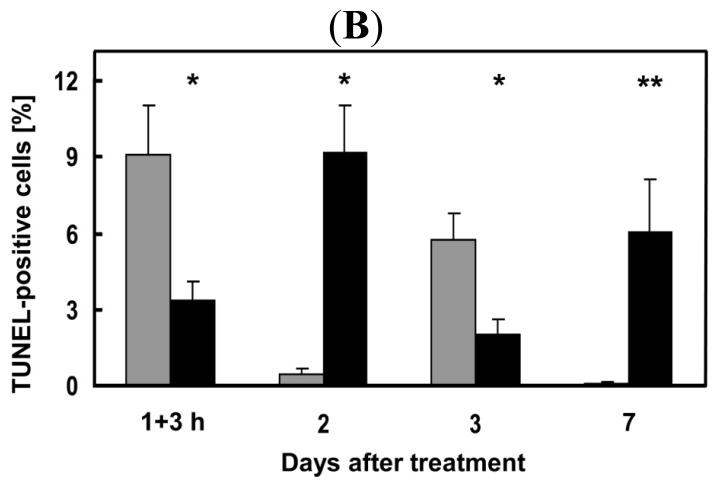
Time course of apoptosis after treatment with APAP. Mice were treated as described in Figure 2. Cells were delivered via intrasplenic injection (+hMSC-HC); control mice received PBS (−hBM-MSC). Three hours later (=1 day + 3 h after treatment), and 2, 3, and 7 days after treatment, livers were excised and tissue sections were processed to detect apoptotic cells by the TUNEL assay. In (**A**) representative pictures of livers are shown at the relevant time points 1 day + 3 h and 2 days after treatment with APAP, Scale bar: 100 μm. Arrowheads indicate TUNEL-positive nuclei; The apoptotic rate (**B**) was calculated as described in Materials and Methods. Values are means ± SEM from 2 to 3 livers (*N* ≥ 2), from which at least five different sections were counted (*n* ≥ 5). Values of controls without hMSC-HC (grey bars) are significantly different from values of livers with hMSC-HC (black bars) at the * *p* ≤ 0.01 or ** *p* ≤ 0.025 level.

The lack of obvious tissue lesions after APAP treatment and subsequent cell transplantation could have two reasons: (i) The transplanted cells attenuated the APAP-caused cell damage and/or (ii) stimulated hepatocyte proliferation, thus minimising tissue deterioration. Perivenous tissue lesions after APAP treatment are due to apoptotic and necrotic cell damage. Therefore, we determined the apoptosis rate by the TUNEL method, as described in Materials and Methods. Three hours (short-term) and 1, 2, and 6 days (long-term) after hMSC-HC transplantation, livers were explanted for histological analyses. Representative microscopic pictures of TUNEL-stained liver sections of 1 day + 3 h and 2 days after APAP treatment are shown in Figure 5A. Three hours after cell transplantation, the apoptotic rate was significantly lower in livers with hMSC-HC as compared to controls without hMSC-HC, which was reversed 2 days after treatment. Seven days after treatment, no apoptosis was detected in control livers but it was still elevated in livers with cell transplants (Figure 5B). Thus, apoptosis was significantly attenuated by hMSC-HC immediately after transplantation (short-term) while at later time points apoptosis was increased in livers with compared to livers without hMSC-HC.

**Figure 6.**
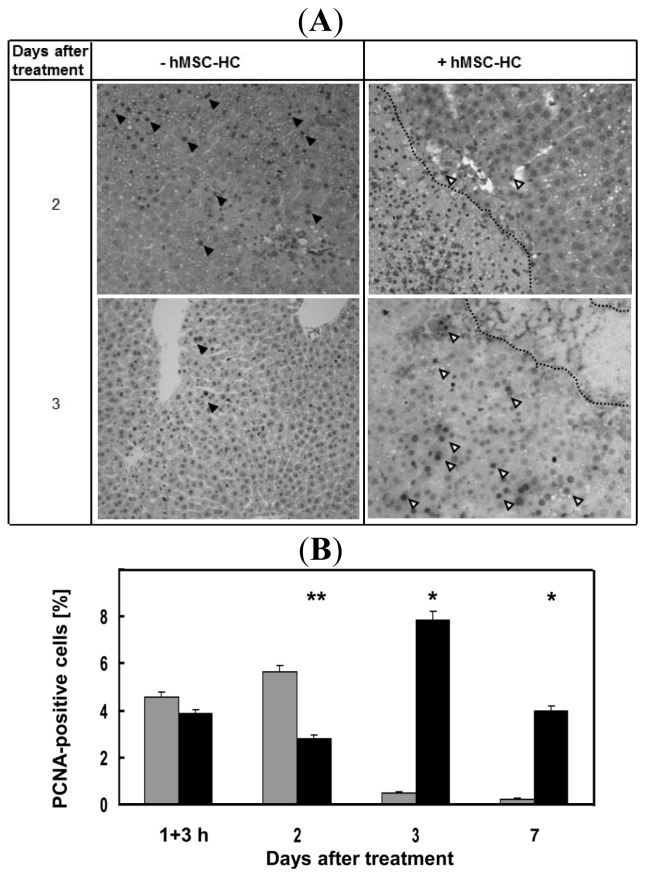
Time course of hepatocyte proliferation after treatment with APAP. Mice were treated as described in the legend to Figure 2. Cells were delivered via intrasplenic injection (+hMSC-HC), control mice received PBS (−hBM-MSC). Three hours later (=1 day + 3 h after treatment), and 2, 3, and 7 days after treatment, livers were excised and tissue sections were processed to detect proliferating cells by immunohistochemical staining of PCNA. In (**A**) representative pictures of livers are shown at the relevant time points 2 and 3 days after treatment with APAP. Solid and open arrowheads indicate PCNA-positive nuclei in stained sections, from livers without and with treatment of hMSC-HC, respectively; The proliferation rate (**B**) was calculated as described in Materials and Methods. Values are means ± SEM from 2 to 3 livers (*n* ≥ 2), from which at least five different sections were counted (*n* ≥ 5). Values of controls without hMSC-HC (grey bars) are significantly different from values of livers with hMSC-HC (black bars) at the * *p* ≤ 0.01 or ** *p* ≤ 0.025 level.

The regenerative response of the liver to APAP treatment was measured by determination of the proliferation rate. The proliferation marker PCNA was stained immunohistochemically and positive nuclei counted as described (Figure 6A). Livers were analysed already 3 h after cell transplantation since we supposed a rapid response of the liver to the cell transplants. Yet, at this point in time, no differences in the proliferation rate between transplanted and control animals were detected. However, 2 days after treatment, proliferation was significantly enhanced in livers of control animals compared with transplanted animals. In contrast, 3 and 7 days after APAP treatment the proliferation rate was significantly higher in transplanted *vs.* control animals (Figure 6B). Thus, the regenerative response seemed delayed in animals receiving hMSC-HC.

### 2.6. Maintenance of Functional Capacity by hMSC-HC after APAP-Induced Liver Injury

**Figure 7.**
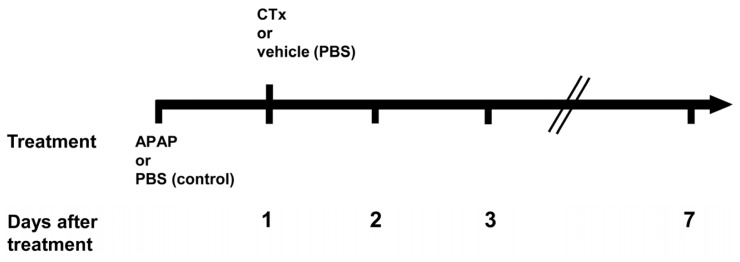
Schematic overview of the experimental design. Animals were injected acetaminophen (APAP) or phosphate-buffered saline (PBS) as control. One day after treatment, partial hepatectomy (PH) followed by cell transplantation (CTx) (or PBS injection as vehicle control) was performed. Samples for biochemical and histological analyses were taken 1, 2, 3, and 7 days after treatment. At day 1, samples were taken just prior to PH and CTx.

**Figure 8.**
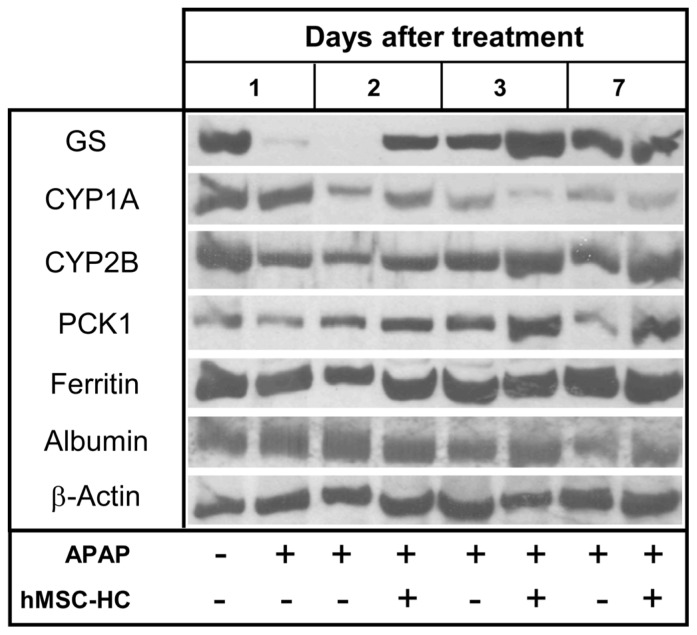
Time course of the expression of functional marker proteins after APAP treatment. Mice were injected APAP as indicated. One day later (=1 day after treatment), one third hepatectomy was conducted and hMSC-HC were delivered via intrasplenic injection (+hMSC-HC) as described in Materials and Methods. Controls received PBS instead of hMSC-HC (−hMSC-MSC). Another 1 day (=2 days after treatment), 2 days (=3 days after treatment) and 6 days (=7 days after treatment) later, livers were harvested and cytosolic extracts were prepared for Western blot analyses. The expression of the perivenous marker enzymes glutamine synthetase (GS) and cytochromes P450 1A and 2B, the periportal marker enzyme phosphoenolpyruvate carboxykinase (PCK1) and the plasma proteins ferritin and albumin were detected using the specific antibodies as listed in Table 3. β-actin served as a loading control. The blots shown represent a series of 2–3 different experiments.

The data so far show that transplanted hMSC-HC after APAP treatment colonised the damaged perivenous areas and, by the attenuation of apoptosis immediately after transplantation (short-term), prevented progress of liver injury. To investigate whether this had functional consequences, the expression of perivenous marker enzymes like GS or members of the cytochrome P450 family such as CYP1A or 2B was determined by Western blotting. In addition, the expression of the periportal marker enzyme phosphoenolpyruvate carboxykinase (PCK1) and of the plasma proteins ferritin and albumin were analysed. Mice were treated with APAP and hMSC-HC were transplanted 24 h later as outlined in Figure 7. Livers were explanted 1, 2, and 6 days after cell transplantation. One day after treatment with APAP, the expression of GS and the CYPs was markedly reduced. Also, the expression of the PCK1 gene seemed slightly reduced indicating progression of liver damage into the periportal areas of the liver. While the expression in control animals further decreased with time progressing, it recovered already at 2 days after APAP treatment and beyond in livers receiving hMSC-HC. Thus, at nearly any point in time after APAP treatment, the expression of the functional marker enzymes was higher in livers with cell transplants than without. The expression of the plasma proteins seemed unaffected by APAP treatment (Figure 8). This indicates that the hMSC-HC attenuated the progress of APAP-induced liver damage and thus maintained hepatic metabolic capacity.

### 2.7. Long-Term Functional Engraftment of hMSC-HC into Mouse Livers after APAP-Induced Injury

**Figure 9.**
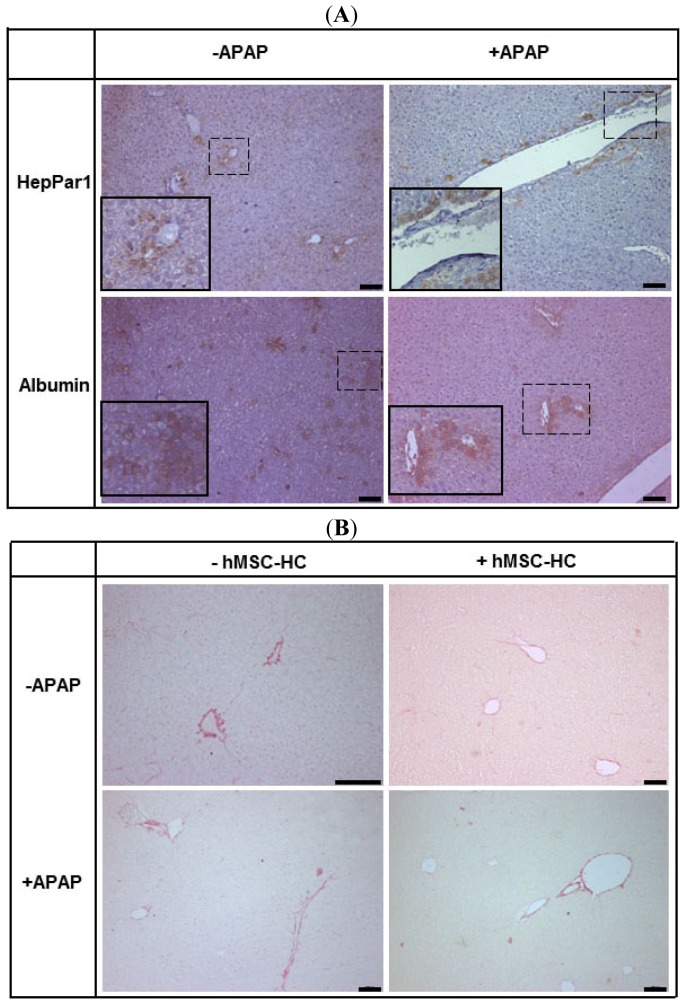
Functional integration of hMSC-HC into mouse livers treated with and not treated with APAP. Animals were treated with APAP (+APAP) or with PBS as a control (−APAP). Twenty-four hours later, hMSC-HC were transplanted into the livers as indicated (+hMSC-HC) and described in Materials and Methods. Controls received PBS instead (−hMSC-HC). Seven weeks later, livers were explanted and human stem cell-derived hepatocytes were detected immunohistochemically in the mouse liver parenchyma by the use of human anti-HepPar1 and human anti-albumin antibodies (**A**). In (**B**), collagen was stained histochemically by the Sirius red method as described in Materials and Methods. Scale bar: 100 μm; the insets in (**A**) show higher magnifications of the dotted areas.

Seven weeks after treatment with APAP, liver histology appeared normal both in animals with and without transplantation of hMSC-HC. Human stem cell-derived hepatocytes in the mouse host liver were identified by staining of human albumin and the human hepatocyte-specific antigen HepPar1 that detects mitochondrial carbamoylphosphate synthetase (CPS I), the key regulatory enzyme of the urea cycle. Integrated cells expressed both human albumin and HepPar1. Irrespective of previous APAP treatment, hMSC-HC were localised in the periportal areas of the liver (Figure 9A). Human albumin was localised both intracellular and in the sinusoids indicating its secretion. Since urea synthesis is a specific function of periportal hepatocytes, expression of carbamoylphosphate synthetase in periportally localised transplanted hMSC-HC indicates regional-specific integration and function of the transplanted cells. Thus, the transplanted hMSC-HC functionally integrated into the mouse liver parenchyma both under control and APAP treatment conditions.

There is evidence that mesenchymal stem cells transplanted into mouse livers might increase fibrosis after acute liver injury [42]. However, staining of liver sections from untreated animals and animals treated with APAP with sirius red did not show significant differences in the collagen content in non-transplanted and transplanted animals (Figure 9B). Thus, hMSC-HC seemed not to provoke fibrosis neither under normal nor acute injury conditions.

### 2.8. Tissue Re-Arrangement after hMSC-HC Transplantation

**Figure 10.**
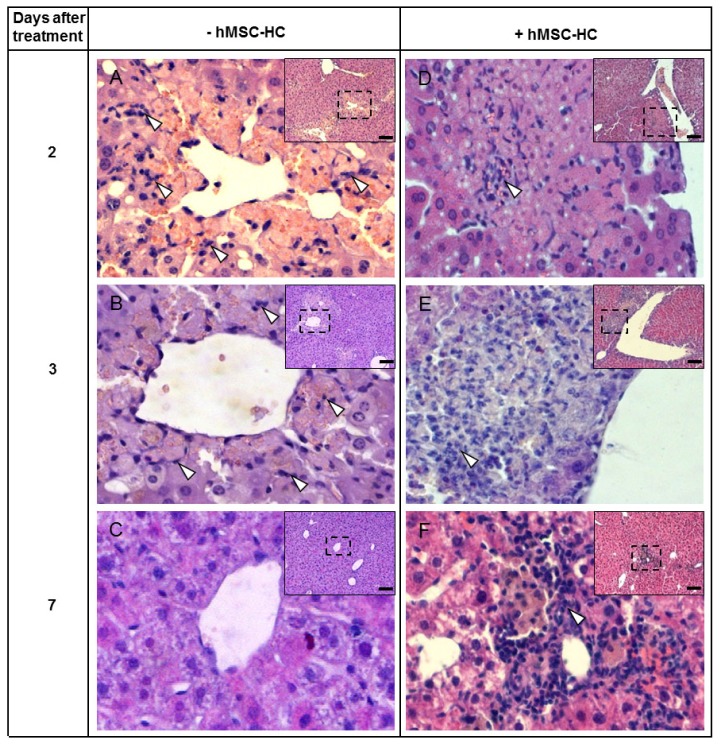
Infiltration of inflammatory cells in livers of mice after APAP treatment. Mice were treated as described in the legend to Figure 2. One day after APAP treatment, one third hepatectomy was conducted and hMSC-HC were delivered via intrasplenic injection (+hMSC-HC, (**D**–**F**)) as described in Materials and Methods; Controls received PBS instead of hMSC-HC (−hMSC-MSC (**A**–**C**)); Another 1 day (=2 days after treatment (**A**,**D**)), 2 days (=3 days after treatment (**B**,**E**)) and 6 days (=7 days after treatment (**C**,**F**)) later, livers were harvested and tissue sections were stained with HE. Pictures are higher magnifications of the dotted areas shown in the insets; In (**A**) and (**B**) single phagocytic cells infiltrated the areas damaged by APAP (arrowheads), which were absent 7 days after treatment (**C**). In livers receiving cell transplants massive infiltrates were visible at any point in time after treatment. Scale bar in the original image: 100 μm.

Comparing the perivenous localisation of transplanted hMSC-HC short-term (Figures 3 and 4) with the periportal localisation long-term (Figure 9A), the question of tissue clearance of the cell transplants arises. We investigated the time course of the appearance of cells of the innate immune system such as macrophages or granulocytes, which are the most likely candidates to clear cell transplants from the perivenous areas. Two days after treatment with APAP, cell infiltrates become visible at the boundaries of the perivenous areas destructed by APAP (Figure 10A). These infiltrates contain macrophages and occasional granulocytes, which are still visible at 3 days, even if at negligible frequency, and have disappeared 7 days after APAP treatment (Figure 10B,C). This time course correlates well with the regeneration of the liver tissue after the acute APAP injury (*cf.* Figure 3B–D) indicating that macrophages and granulocytes are involved in phagocytosis of cell garbage during regeneration. hMSC-HC transplants were visible in perivenous areas after APAP treatment (Figure 4). These were highly infiltrated by macrophages and granulocytes at 2 and 3 days after APAP treatment and were still clearly visible 7 days after treatment (Figure 10D–F). Abundance of phagocytic cells was significantly higher as in livers without cell transplantation indicating the stimulation of recruitment by the hMSC-HC transplants. Since damage of perivenous hepatocytes was hindered by hMSC-HC, it is obvious that phagocytic infiltrates were not involved in the clearance of damaged hepatocytes but rather the transplanted cells.

## 3. Discussion

### 3.1. Histopathologic Impact of hMSC-HC on Liver Regeneration after APAP Injury

The expression of enzymes of the cytochrome P450 system is higher in perivenous compared to periportal hepatocytes, which makes the perivenous hepatocytes more vulnerable to acetaminophen. The drug is either conjugated with sulfate or glucuronate and eliminated, or converted by cytochrome P450-dependent oxidation to *N*-acetyl-*p*-benzoquinonimine (NAPQI), which is secreted into and eliminated with the bile after conjugation to glutathione. Even if only less than 10% of APAP is metabolised via this way [43,44], the final harmful consequence of acetaminophen overdosage is the depletion of glutathione and thus the lack of protection against oxidative stress and lipid and protein modification by covalent binding of NAPQI. Mitochondrial damage seems to be the initial event fostering hepatocyte apoptosis, which in turn perpetuates and finally causes massive necrosis of perivenous hepatocytes [45–48]. Besides these initial effects of APAP-induced hepatotoxicity, pro-inflammatory cytokines like TNFα and IL1β are elevated after APAP treatment and may trigger an inflammatory response, thus potentially augmenting tissue damage [49–51]. Since mesenchymal stem cells display anti-inflammatory and pro-regenerative features [15,17,47,52], we reasoned that transplantation of MSC into APAP-treated livers might alleviate liver injury. It has been shown for stem cell-derived hepatocytes that after portal injection they are entrapped in the proximal branches of the portal vein and eventually traverse the endothelia to integrate long-term into the portal parenchyma featuring the same qualities as their neighbouring periportal hepatocytes [20,27,53]. This was corroborated in the present study (Figures 2 and 9). Yet, in livers treated with APAP, transplanted cells were initially found in the perivenous regions of the liver parenchyma (Figures 3 and 4). It may only be speculated that this was due to a low pressure region generated by APAP-induced hepatocyte necrosis in the perivenous parenchyma facilitating flow of transplanted cells from the proximal into the distal parts of the sinusoids. This, however, needs confirmation. It is also conceivable that the stem cell transplants actively invaded the necrotic tissue due to the migratory features of mesenchymal stem cells. MSC have been shown to home to the mouse liver with ischemia/reperfusion injury more efficiently, when Notch signalling was impaired as compared to wildtype MSC, which was likely due to the suppression of CXCR4 expression regulating chemotaxis towards SDF-1 [54]. In mouse models of liver cirrhosis induced by either CCl_4_ or thioacetamide treatment, the knock-down of the androgen receptor improved migration as well as anti-inflammatory and anti-fibrotic actions thus augmenting liver repair [55]. Hepatocytic differentiation of MSC prior to transplantation yielded higher repopulation of the host liver compared to native MSC indicating priming of the cells for better integration [53].

Transplanted hMSC-HC residing in the perivenous areas of the liver seemed to attenuate the progress of APAP-induced tissue damage (Figure 3), corroborated also by inhibition of apoptosis at early points in time after APAP treatment (Figure 5). The rate of apoptosis is significantly increased in a biphasic manner indicating a direct deleterious effect of APAP on hepatocytes at early and the re-arrangement of the liver tissue architecture at later points in time after APAP treatment. These peaks are postponed in livers with MSC transplants. Since no obvious tissue damage by APAP is observed in these livers, it may be anticipated that tissue repair occurs only in the time and physical frame needed to replace MSC transplants by hepatocytes emerging from self-regeneration of the liver parenchyma. Taken together, the results show that hMSC-HC protected the mouse liver from APAP-induced liver failure by attenuating tissue damage through their anti-inflammatory and anti-apoptotic action. In NOD-SCID mice, both native and hepatocyte-differentiated MSC from bone marrow [23] and amniotic fluid [56] contributed to liver regeneration after CCl_4_ treatment inducing fulminant hepatic failure. Hepatic regeneration was stimulated as well as metabolic and inflammatory stress reduced by adipose tissue-derived MSC in rat livers treated with acetaminophen [57]. Thus, in line with these reports, our results confirm the liver-protective features of transplanted MSC in the setting of acute liver failure.

### 3.2. How Do hMSC-HC Ameliorate Liver Damage Caused by APAP?

To say it frankly at the beginning: We do not really know the answer to this question. One thing is very clear. Even if hepatocyte-differentiated hMSC were used in the present study, it must be anticipated that the time frame, in which improvement of the liver from APAP damage occurred, was too short to be mediated by functional metabolic support of fully integrated cell transplants. This assumption is in line with our previous findings from hepatocyte as well as stem cell transplantation experiments showing that most of the cells were cleared from the host liver in the course of the first days post-transplant. Functionally integrated cells are seen only weeks after transplantation [20,27,58,59]. As shown in Figure 9, cells indeed integrated into the parenchyma after 7 weeks and executed hepatocyte-specific functions like albumin secretion and probably urea synthesis as verified by the expression of HepPar 1, which has been shown to be identical to carbamoylphosphate synthase [60], the entry enzyme of the urea cycle. Yet, this represents only a small portion of the cells initially transplanted ranging in the order of magnitude of 1% as shown previously [41]. Since at that, and long before that point in time the liver had completely recovered from APAP-induced injury, metabolic support provided by transplanted stem cells seems to play only a minor role in preventing acute liver damage shortly after intoxication. These results corroborate findings from other groups showing that irrespective of whether acute liver injury was induced periportally by allylalcohol [61] or perivenously by CCl_4_ [22,62,63] or acetaminophen [47], MSC-derived hepatocyte-like cells repopulated the diseased host liver, though at a low rate of about 1% of the total liver mass.

If not substantial provision of metabolic capacity is the key action of MSC-derived hepatocytes to support liver regeneration after acute injury, it seems reasonable to suppose that paracrine factors are the players. Indeed, d-galactosamine-induced acute liver failure was ameliorated in rats by the inhibition of apoptosis and stimulation of hepatocyte proliferation mediated by MSC-derived factors [25,26]. The paracrine mode of action has also been demonstrated in another mouse model of whole body irradiation. Human MSC applied systemically were found in the liver potentially mediated by mir-27b down-regulation in liver and up-regulation of SDF1 expression. hMSC decreased oxidative stress as shown by reduction of apoptotic cells and up-regulation of *Nrf2*, *SOD* gene expression, which could also promote liver regeneration corroborated by the up-regulation of growth factors NGF, HGF, and anti-inflammatory molecules IL-10, IL1-RA mRNA. Furthermore, MSC could support endothelial function, since hMSC expressing VEGF and Ang-1 were found in the perivascular region, which coincided with an increased expression of VEGFr1, r2 in the liver [40]. Pinpointing also to a paracrine systemic action, MSC attenuated LPS- or burn-induced systemic inflammation [64]. Supporting the notion of systemic paracrine actions, MSC were mobilised from the bone marrow and homed into the liver after acute intoxication with CCl_4_ or 2-acetylaminofluorene, which also indicated the migratory features of MSC [65,66]. Communication between transplanted MSC and host tissue cells has also been shown to occur by MSC-derived exosomes allowing to exchange mRNA and miRNA between donor and host cells [67,68].

Initially, we anticipated that the liver after acetaminophen poisoning needed metabolic support until start of regeneration. Therefore, we used MSC prior differentiated into hepatocyte-like cells. This, however, turned out not to be the major mode of action. Instead, we saw transplanted cells occupying the destroyed perivenous areas of the liver lobule, obviously preventing disease progression by paracrine mechanisms, thus corroborating previous results with undifferentiated MSC as discussed above. Therefore, we would expect an even more pronounced positive impact on tissue regeneration when using native MSC. In summary, the data show that MSC and MSC-derived hepatocyte-like cells feature a broad pleiotropic pattern of actions, which, however, might be different in different disease settings. We are far from understanding the molecular nature of these various actions, which thus remain to be identified unequivocally under each experimental condition.

It is without doubt that biochemical reactions as described above resulting in oxidative stress, protein and lipid modification, and mitochondrial dysfunction are hallmarks in the initiation of APAP-induced acute liver injury. However, it becomes more and more obvious that besides the mechanisms affecting primarily the individual hepatocytes metabolising the drug, non-hepatocyte liver cells or inflammatory cells invading the liver are important players in the pathogenesis of acute liver failure. Necrotic cell death results in the liberation of damage-associated molecular patterns (DAMPs) comprising besides others e.g., DNA fragments and high mobility group box 1 protein (HMGB1) released from the perishing hepatocytes. These may activate liver-resident macrophages, the Kupffer cells, to secrete pro-inflammatory cytokines and chemokines, though these seem not to be primarily involved in the perpetuation of liver damage but rather in the recruitment of circulating inflammatory cells like granulocytes and macrophages [69,70]. In this study, we observed the massive recruitment of phagocytic cells in livers receiving cell transplantation after APAP treatment (Figure 10). Accumulation of these cells was dominant in regions adjacent to the cell transplants, which is simultaneously the boundary to the surrounding liver tissue. They persisted even at 7 days after APAP treatment, at which point in time the histologic aspect of the liver seemed normal again. It is well feasible to suppose that the inflammatory infiltrates were involved in the phagocytosis of both perishing cell transplants and hepatocytes damaged by APAP. Clearance of necrotic cells has been claimed one prominent prerequisite for liver regeneration after APAP injury [71]. This concept is supported by our observation that shortly after cell transplantation, tissue damage is attenuated as demonstrated histologically (Figure 3) and by the inhibition of apoptosis (Figure 5) but at later points in time, tissue re-arrangement by clearance of cell transplants and damaged hepatocytes by infiltrating macrophages is achieved as regeneration progresses.

## 4. Materials and Methods

### 4.1. Isolation, Propagation and Hepatocyte Differentiation of Human Bone Marrow-Derived Mesenchymal Stem Cells (hBM-MSC)

Human BM-MSC were obtained from femur bone marrow (hBM) waste material, collected after elective knee and hip joint surgery, from donors who had given written consent according to the permission of the Institutional Ethics Review Board of the Medical Faculty of the Martin-Luther University of Halle-Wittenberg (Halle, Germany). The bone marrow was collected in citrate buffer and processed as described previously [20]. Briefly, the mononuclear cell fraction was collected by density gradient centrifugation and single cells were plated on uncoated tissue culture flasks at a density of 1 × 10^5^ cells/cm^2^. Upon confluence, cells were harvested by conventional trypsin/EDTA treatment, re-seeded at a density of 100–200 cells/cm^2^ and cultured as described [20]. At 80%–90% confluence, hepatocyte differentiation was initiated by a demethylation step using 5′-azacytidine (Sigma-Aldrich, Munich, Germany) for 24 h. Thereafter, the medium was changed to hepatocyte differentiation medium as detailed previously. Cells were cultured for another 14 days replacing medium every 4 days. During the culture, cells changed their morphology from fibroblastoid into polygonal and acquired typical features of differentiated hepatocytes like urea and glycogen synthesis as well as expression of prominent hepatocyte proteins like albumin, phosphoenolpyruvate carboxykinase, glutamine synthase, cytochrome P450 enzymes and carbamoylphosphate synthase [41]. Cells were not further passaged or amplified and, after detachment from the dishes, were directly used for transplantation as outlined below.

### 4.2. Acetaminophen Treatment of Immunodeficient Mice and Transplantation of hBM-MSC

All animal procedures were performed in accordance with the German legislation for animal protection. Animals were kept under a 12 h day and night rhythm and received a standard rodent diet *ad libitum* with free access to water. To restrain liver regeneration by host hepatocytes, and thus providing a growth advantage to transplanted cells, male *Pfp/Rag2^−/−^* mice (20–25 g; Taconic, Ejby, Denmark) at the age of 8–12 weeks were treated with propranolol hydrochloride (60 mg/kg body weight dissolved in drinking water; Schwarz Pharma, Monheim, Germany) for 3 days before cell transplantation [72]. Dissolved acetaminophen (735 mg in PBS/kg body weight if not otherwise indicated; Sigma-Aldrich, Munich, Germany) or vehicle solution (Phosphate-buffered saline, PBS) as control were injected intraperitoneally 24 h before cell transplantation. Both acetaminophen administration and the corresponding PBS control are referred to as “treatment” in the following. The cell transplantation procedure was initiated by one third partial hepatectomy to provide a mitotic challenge to the liver. Eventually, 1 × 10^6^ hepatocyte-like cells pre-differentiated from hBM-MSC *in vitro* with a vitality of >75% were administered in a volume of 75 μL by intrasplenic injection [41]. Control animals received PBS instead of cells. An overview of the experimental design and the time schedule applied is given in Figure 7. Livers were harvested at the times indicated and then either snap frozen and stored at −80 °C, or fixed in 4% Histofix (Roth, Karlsruhe, Germany) for further histological analysis.

### 4.3. Histological Procedures

#### 4.3.1. HE Staining

HE staining was performed according to standard histological protocols. After dewaxing, slices were incubated with haematoxylin for 2 min and thereafter stained with eosin. Dehydrated slices were embedded in Entellan^®^ (Merck, Darmstadt, Germany) for microscopic analysis.

#### 4.3.2. TUNEL-Assay

To detect apoptotic cells, the ApopTag Plus Peroxidase *In Situ* Apoptosis Detection Kit from Millipore (Merck Millipore, Darmstadt, Germany) was applied as described in the manual. The percentage amount of TUNEL-positive cells was estimated by counting the number of stained cells in randomly defined microscopic areas and dividing by the total number of cells.

#### 4.3.3. Immunohistochemistry

Paraffin slices (1 μm) were dewaxed and sequentially incubated in citrate buffer (pH 6.0) for 30 min, H_2_O_2_ (3%) solution for 30 min, blocking solution (5% BSA and 0.5% Tween 20 in PBS) for 1.5 h, and finally avidine/biotin blocking (Vector Laboratories, Burlingame, CA, USA) solution for 30 min. Subsequently, the slices were incubated with the primary antibody overnight at 4 °C. After three washings with PBS, 30 min incubation with the secondary antibody at room temperature followed. After three washing steps with PBS, the ABC-kit (only used for biotinylated secondary antibody) and DAB substrate kit for peroxidase (both from Vector Laboratories) were used according to the manufacturer’s manuals. The respective primary and secondary antibodies used and their dilutions are listed in Table 2.

To detect proliferating cells, the percentage amount of PCNA-positive nuclei was estimated by counting the number of stained nuclei in randomly defined microscopic areas and dividing by the total number of nuclei.

#### 4.3.4. Sirius Red Staining

Collagen fibres were detected by staining with sirius red. After dehydration in a descending ethanol series, the slides were incubated with sirius red solution (0.5 g direct red 80 (Alfa Aesar, Karlsruhe, Germany) per 500 mL of 1.3% picric acid solution (Fluka, Darmstadt, Germany) for 1 h). Thereafter, slides were washed twice with 0.5% acetic acid, dried and then mounted in Entellan^®^ for further microscopic analysis.

### 4.4. Tools and Assays

#### 4.4.1. Determination of Serum Transaminase Activity

The activity of aspartate aminotransferase (AST) was determined spectrophotometrically in the mouse sera from blood samples taken at the times indicated in Figure 7. The samples were analysed by the central laboratory at the University Hospital of Halle (Halle, Germany).

#### 4.4.2. Western-Blotting

Protein content of liver cytosolic extracts was quantified by the method of Bradford [73]. A total of 25 μg protein per lane was loaded onto a 10% SDS polyacrylamide gel and separated by electrophoresis. Proteins were transferred to a polyvinylidene fluoride (PVDF; Roth, Karlsruhe, Germany) membrane by the semi-dry blotting method. After blocking with buffer (200 mM Tris, 1.5 M NaCl, 1% Tween 20 at pH 7.5) containing 5% milk powder, membranes were incubated with the primary antibodies at 4 °C overnight. Following three washings for 10 min each in buffer containing 1% milk powder, the membranes were incubated with the secondary antibody for 1 h at room temperature. The membranes were then again washed three times and specific protein bands were visualized by the ECL reagent (GE Healthcare, Munich, Germany) according to the supplier’s manual. Primary and secondary antibodies and their dilutions are listed in Table 3.

#### 4.4.3. Statistics

Significant differences were verified by the Student’s *t*-test for unpaired values (*n* ≥ 3) at the *p*-level as given in the legends to the figures.

## 5. Conclusions

Human MSC pre-differentiated into hepatocyte-like cells infiltrate perivenous liver areas, which are the primary tissue targets of damage caused by acetaminophen. They might attenuate progression of liver injury short-term by as yet unknown mechanisms. Repair and re-building of liver architecture after APAP treatment seems prolonged in livers where hMSC have been transplanted in the long-term range, which is nevertheless compatible with improvement of metabolic functions. Thus, MSC support functional liver regeneration after acute liver injury and might be of high therapeutic interest in future clinical settings.

## Figures and Tables

**Table 1.** Time-dependent increase in aspartate aminotransferase (AST) activity in the serum of mice treated with 735 mg/kg acetaminophen (APAP).

Days after treatment	AST activity [μkat/L]			

	PBS	PBS + hMSC-HC	APAP	APAP + hMSC-HC
1	1.07	n.d.	141.92 ± 19.46	n.d.
2	8.96 ± 4.09	11.12 ± 4.64	18.46 ± 2.07	80.80 ± 13.49
3	1.63	4.32 ± 0.29	2.42	8.09 ± 2.95
7	1.54	1.05 ± 0.12	0.90	1.34 ± 0.07

Control mice were treated with phosphate-buffered saline (PBS). Values are means ± SEM from sera of 2–4 animals; hMSC-HC, hepatocyte-differentiated human bone marrow-derived mesenchymal stem cells. n.d., not determined (same values as 1 day after treatment with PBS and APAP, respectively).

**Table 2.** Antibodies and dilutions used for immunohistochemistry.

Antigen	Primary antibody			Secondary antibody		
	
	Company	Species	Dilution	Company	Species	Dilution
PCNA	Genetex (Aachen, Germany)	mouse-anti mouse	1:250	Vector Laboratories (Burlingame, CA, USA)	Biotin-conjugated horse anti-mouse IgG	1:200

HepPar1	Dako (Hamburg, Germany)	Monoclonal mouse anti-human hepatocyte clone OCH1E5	1:50	BD (Heidelberg, Germany)	HRP-conjugated goat anti-mouse IgG	1:200

Albumin	Abcam (Cambridge, UK)	polyclonal rabbit anti-human	1:1000	Dianova (Hamburg, Germany)	biotin-conjugated donkey anti-rabbit	1:200

Glutamine synthetase	BD (Heidelberg, Germany)	monoclonal mouse anti-mouse, human	1:1000	Dianova (Hamburg, Germany)	HRP-conjugated donkey anti-mouse	1:200

HRP, horseraddish peroxidase; IgG, Immunoglobulin G.

**Table 3.** Antibodies and dilutions used for Western blot analyses.

Antigen	Primary antibody			Secondary antibody		
	
	Company	Species	Dilution	Company	Species	Dilution
Glutamine synthetase	BD (Heidelberg, Germany)	mouse anti-human, rat, mouse	1:6000	BD (Heidelberg, Germany)	HRP-conjugated anti-mouse	1:8000
CYP1A1	Natutec (Frankfurt, Germany)	goat anti-rat, human	1:2000	Dianova (Hamburg, Germany)	HRP-conjugated anti-goat	1:9000
CYP2B	Natutec (Frankfurt, Germany)	goat anti-rat	1:2000	Dianova (Hamburg, Germany)	HRP-conjugated anti-goat	1:9000
PCK	Aviva Systems Biology (San Diego, CA, USA)	rabbit anti-human, mouse, rat, dog	1:700	BD (Heidelberg, Germany)	HRP-conjugated anti-rabbit	1:7500
Ferritin	Self-made	rabbit anti-human	1:3000	BD (Heidelberg, Germany)	HRP-conjugated anti-rabbit	1:7500
Albumin	Dunn Labortechnik (Asbach, Germany)	goat anti-human	1:450	Dianova (Hamburg, Germany)	HRP-conjugated anti-goat	1:9000
β-Actin	Merck Millipore (Darmstadt, Germany)	mouse anti-human, rat, mouse	1:5000	BD (Heidelberg, Germany)	HRP-conjugated anti-mouse	1:10,000

CYP, cytochrome P450 subtype; PCK, cytosolic phosphoenolpyruvate carboxykinase; HRP, horseraddish peroxidase.
